# Ex vivo susceptibility and genotyping of *Plasmodium falciparum* isolates from Pikine, Senegal

**DOI:** 10.1186/s12936-017-1897-6

**Published:** 2017-06-14

**Authors:** Aminata Mbaye, Amy Gaye, Baba Dieye, Yaye D. Ndiaye, Amy K. Bei, Muna Affara, Awa B. Deme, Mamadou S. Yade, Khadim Diongue, Ibrahima M. Ndiaye, Tolla Ndiaye, Mouhamed Sy, Ngayo Sy, Ousmane Koita, Donald J. Krogstad, Sarah Volkman, Davis Nwakanma, Daouda Ndiaye

**Affiliations:** 1 Laboratory of Parasitology/Mycology HALD, Cheikh Anta Diop University of Dakar, PO Box 5005, Dakar, Senegal; 2 Department of Immunology and Infectious Diseases, Harvard School of Public Health, Boston, MA USA; 3 Tulane University, New Orleans, LA USA; 4 Medical Research Council Unit, The Gambia, Fajara, Gambia; 5 University of Bamako, Bamako, Mali

**Keywords:** Chemosensitivity, Genotyping, *Plasmodium falciparum*, Pikine

## Abstract

**Background:**

The monitoring of *Plasmodium falciparum* sensitivity to anti-malarial drugs is a necessity for effective case management of malaria. This species is characterized by a strong resistance to anti-malarial drugs. In Senegal, the first cases of chloroquine resistance were reported in the Dakar region in 1988 with nearly 7% population prevalence, reaching 47% by 1990. It is in this context that sulfadoxine–pyrimethamine temporarily replaced chloroquine as first line treatment in 2003, pending the introduction of artemisinin-based combination therapy in 2006. The purpose of this study is to assess the ex vivo sensitivity to different anti-malarial drugs of the *P. falciparum* population from Pikine.

**Methods:**

Fifty-four samples were collected from patients with non-complicated malaria and aged between 2 and 20 years in the Deggo health centre in Pikine in 2014. An assay in which parasites are stained with 4′, 6-di-amidino-2-phenylindole (DAPI), was used to study the ex vivo sensitivity of isolates to chloroquine, amodiaquine, piperaquine, pyrimethamine, and dihydroartemisinin. High resolution melting was used for genotyping of *pfdhps*, *pfdhfr*, *pfmdr1*, and *pfcrt* genes.

**Results:**

The mean IC_50_s of chloroquine, amodiaquine, piperaquine, dihydroartemisinin, and pyrimethamine were, respectively, 39.44, 54.02, 15.28, 2.23, and 64.70 nM. Resistance mutations in *pfdhfr* gene, in codon 437 of *pfdhps* gene, and an absence of mutation at position 540 of *pfdhps* were observed. Mutations in codons K76T of *pfcrt* and N86Y of *pfmdr1* were observed at 51 and 11% population prevalence, respectively. A relationship was found between the K76T and N86Y mutations and ex vivo resistance to chloroquine.

**Conclusion:**

An increase in sensitivity of isolates to chloroquine was observed. A high sensitivity to dihydroartemisinin was observed; whereas, a decrease in sensitivity to pyrimethamine was observed in the parasite population from Pikine.

## Background

Malaria is a parasitic disease which was responsible for nearly 429,000 deaths worldwide in 2015. More than 92% of these deaths occur in Africa in children [1]. Children under 5 years and pregnant women are among those most vulnerable to malaria. This can be explained by the fact that in malaria-endemic areas, after years of exposure, non-sterilizing immunity develops that is protective against severe malaria. Pregnant women tend to lose this acquired immunity due to the immune-suppression which occurs during pregnancy, and studies have shown that placental parasitaemia in these individuals is higher than those of peripheral blood [2], putting both mother and child at high risk. Many tools are currently available to fight malaria in these vulnerable groups such as intermittent preventive treatment of pregnant women with sulfadoxine–pyrimethamine (SP) [3, 4] and seasonal malaria chemoprevention (SMC) for children under 5 years of age in areas with seasonal transmission of malaria [5]. Currently in Senegal, for SMC in children, the drug regimen of choice is SP-amodiaquine. Amodiaquine is also used in combination with artesunate for treatment of uncomplicated malaria. Cross-resistance has been observed between amodiaquine and chloroquine. Thus, it would be important to monitor the sensitivity of parasites to SP to ensure the effectiveness of these preventive combination treatments. A good correlation between in vivo resistance to SP and in vitro resistance has been characterized as well as a strong association between in vivo resistance and single nucleotide polymorphisms in *pfdhps* (*Plasmodium falciparum* dihydropteroate synthetase) and *pfdhfr (P. falciparum* dihydrofolate reductase) genes [6–10]. I*n* vitro resistance to pyrimethamine is associated with the mutation at codon S108N of *pfdhfr* gene, whereas the resistance to sulfadoxine is associated with the mutation K540E of the *pfdhps* gene. In West Africa, triple mutations at codons N51I, C59R and S108N of the *pfdhfr* gene and the mutation G437A/T in the *pfdhps* gene are frequently observed. In Senegal, the quadruple mutation representing the triple mutation of the *pfdhfr* gene plus the mutation G437A/T in the *pfdhps* is also frequently observed [11–13]. In East Africa, a further mutation in *pfdhps* at codon K540E has been described [9]. This quintuple mutation is highly associated with a therapeutic failure to SP. WHO recommends that in areas where the quintuple mutation reaches greater than 50% population prevalence that SP use should be abandoned for chemoprevention of malaria [2].

The monitoring of amodiaquine, chloroquine, piperaquine, and dihydroartemisinin sensitivity is a clear priority in the fight against malaria. As amodiaquine is used for prevention and treatment of malaria in Senegal, it is important to determine if there is a decrease of sensitivity of *P. falciparum* population to this drug and if this decrease is related to the past (cross-resistance between amodiaquine and chloroquine) or current amodiaquine use. The overall objective of this study is to assess the ex vivo sensitivity of *P. falciparum* isolates from Pikine to SP, amodiaquine, chloroquine, piperaquine, and dihydroartemisinin.

## Methods

### Sample collection

In 54 children aged between 5 and 20 years of age who came for consultation at the Deggo health centre in Pikine in 2014, both venous blood and filter paper were collected. These children suffered from non-complicated malaria with confirmation by drop thick and thin smears. Informed consent by the child and or guardian was requested before any samples were taken. The study protocol was validated by the Human Subjects Committee of Tulane University and the Ethics Committee of the Ministry of Health of Senegal. The work is funded by the International Centres of Excellence for Malaria Research, (ICEMR) West Africa (U19AI089696).

### Ex vivo assays

#### Drug preparation

Pyrimethamine (Sigma), chloroquine diphosphate salt, amodiaquine hydrochloride, dihydroartemisinin, and piperaquine were reconstituted with dimethyl sulfoxide (DMSO). The dilution was performed with the non-supplemented Roswell Park Memorial Institute Medium (RPMI). Twofold serial dilutions were performed with the non-supplemented Roswell Park Memorial Institute Medium (RPMI). The highest drug concentrations plated were 750 nM for chloroquine, 500 nM for piperaquine, 100 nM for amodiaquine, 50 nM for dihydroartemisinin, and 295,056 nM for pyrimethamine. Each drug concentration was plated in duplicate. Plates were frozen at −20 °C until required.

#### Culture and CI50 determination

The tubes of venous blood collected for DAPI test were transported to Aristide Le Dantec Hospital within 6 h of blood draw. The plasma was removed by centrifugation (2500*g* for 10 min). The pellet was then washed twice with unsupplemented RPMI by centrifugation at 2500*g* for 5 min. Parasitaemia was adjusted to between 0.4 and 1% and haematocrit was adjusted to 2%. The parasitaemia and haematocrit adjusted parasite mixture was distributed on the previously dosed 96-well drug plates and incubated in the presence of gas (94% N_2_, 5% CO_2_, 1% O_2_) [14] at 37 °C. After 48 h of incubation, the growth of parasite in positive control wells specifically plated for microscopic evaluation was checked. The assays was determined to be complete when parasites had re-invaded as new rings. Plates were frozen at −20 °C until reading and reading was performed for all plates at once.

For staining and reading the DAPI assay, 100 µl of membrane lysis buffer containing the molecule DAPI was distributed to each well. After a 30 min incubation, plates were centrifuged 4000*g* for 10 min, washed with PBS, and fluorescence was measured using a Fluoroskant Ascent. IC_50_ values were calculated using graph Pad Prism software version 5. Reference clone 3D7, sensitive to all anti-malarial drugs tested, was used for each batch of drug plates as a positive control.

#### DNA extraction and single nucleotide polymorphism typing

Parasitic DNA was extracted using the QIAamp DNA Blood Mini kit (Qiagen) according to manufacturer instructions. Codons 51, 59–108 of the *pfdhfr* gene, 436, 437, 540, and 613 of *pfdhps*, 76 of *pfcrt* and 86 of *pfmdr1* were genotyped by HRM [12]. Glass capillaries were used with a 10 µl final volume. All PCR were performed using 2.5X LightScanner master mix (Biofire), with forward primers at a final concentration of 0.05 µM, reverse primers at a final concentration of 0.2 µM (asymmetric PCR), allele specific probes at a final concentration of 0.2 µM, and 1 µl of genomic DNA, as previously described [12]. Standard software included with the instruments was used for unlabelled probe analysis to visualize melting peaks based on different melting temperatures, indicative of different base pairs, and compared with controls to call alleles for a given assay.

#### Statistical analysis

The Graph-pad Prism Software version 5 was used to calculate the IC_50_ value for all drugs for each parasite isolate tested. For each codon position, the distribution of IC_50_s were compared using the Mann–Whitney U test. The test is significant if the P value is less than 0.05.

## Results

### Ex vivo sensitivity to chloroquine, amodiaquine, piperaquine, dihydroartemisinin, and pyrimethamine

**Fig. 1:**
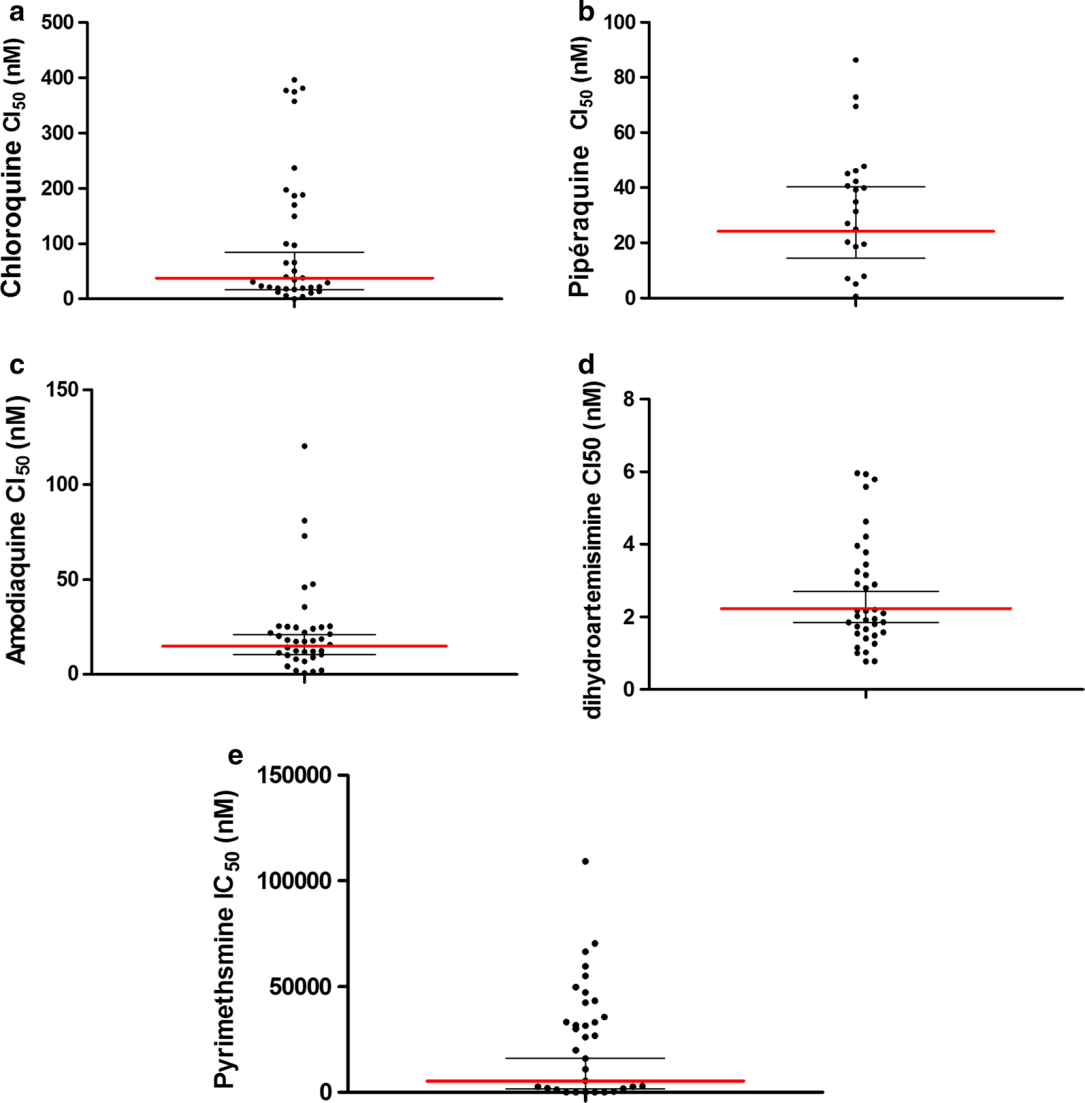
Distribution of IC_50_ value among parasites collected in Pikine in 2014 and tested again Chloroquine (**a**), Piperaquine (**b**), Amodiaquine (**c**), Dihydroartemisinin (**d**) and Pyrimethamine (**e**).* Horizontal lines* indicate the geometric mean of IC_50_ value in* red* with the 95% CI. For *all panels* IC_50_ high value is not represented

Good sensitivity of the 3D7 reference strain to chloroquine (22.05 nM) was observed. The geometric mean IC_50_ for all isolates for chloroquine, amodiaquine, piperaquine, dihydroartemisinin, and pyrimethamine were 35.44, 54.02, 15.28, 2.23, and 64.70 nM, respectively (Fig. 1).

### Prevalence of point mutations of *pfdhps, pfdhfr, pfcrt,* and *pfmdr1* genes

Mutations at codons 51, 59 and 108 of dhfr were highly prevalent with 100, 95 and 96% observed, respectively. For codon 437, a proportion of 44% with mixed wild type and mutant (2%) was observed. No mutation was observed at the 540 position of *pfdhps* gene. For codon 76 of the *pfcrt* gene 51% of mutations and 6% of mixed was found. About the codon 86 of *pfmdr1*, the mutation rate amounted to 11% (Table 1).

**Table 1:** Prevalence of point mutation of *pfdhps*, *pfdhfr*, *pfcrt*, and *pfmdr1* genes at positions 51, 59, 108, 437, 540, 76, and 86

Gene	Codon	Allele	Prevalence
*Pfdhfr*	N51I	I	100% (51/51)
	C59R	R	96% (49/51)
	S108N	N	98% (50/51)
		Mixte	2% (1/51)
*Pfdhps*	A437G	G	44% (24/54)
		G/A	2 (1/54)
	K540E	K	100% (54/54)
		E	0%
*Pfcrt*	K76T	T	51% (28/540)
		Mixte	6% (3/54)
*Pfmdr1*	N86Y	Y	11%
		Mixte	0%

All strains that contained the mutation N86Y of *pfmdr1* gene were mutant or mixed at codon K76T of *pfcrt*. For *pfdhps* and *pfdhfr* triple, quadruple and quintuple mutation were observed at 95, 39 and 0% prevalence, respectively (Table 2).

**Table 2:** Prevalence of mutant haplotype in pfdhps and pfdhfr

Haplotype	Prevalence
Triple mutation	95% (40/42)
Quadruple mutation	39% (15/38)
Quintuple mutation	0% (0/44)

### Association between genotype and phenotype

The ex vivo resistance to chloroquine has been found to be linked to the mutation at codon K76T of *pfcrt* gene and N86Y of *pfmdr1* (Table 3). For amodiaquine, the observation was that the values found for the mutant strains on these codons were higher but not significantly different. In our study, no relationship was found between the mutation K76T and N86Y and the decrease in ex vivo sensitivity to piperaquine, dihydroartemisinin and pyrimethamine. High prevalent of mutation on codon 51, 59 and 108 of the *pfdhfr* gene was found, probably related to the decrease in sensitivity to pyrimethamine.

**Table 3:** Correlation between mutation at *Pfcrt* codon K76T and *Pfmdr1* codon N86Y and sensitivity to chloroquine, amodiaquine, piperaquine, dihydroartemisinin, and pyrimethamine

Compound	Codon	GM of IC50 (nM) for WA	GM of IC50 (nM) for MA	P value
Chloroquine	K76T	13.86	74.15	0.0195
	N86Y	33.90	391.2	0.0279
Amodiaquine	K76T	9.924	10.47	0.0539
	N86Y	9.417	20.69	0.5290
Piperaquine	K76T	56.16	46.45	0.9370
	N86Y	27.15	58.00	0.8155
Dihydroartemisinin	K76T	2.139	1.773	0.4614
	N86Y	2.165	1.906	0.7727
Pyrimethamine	K76T	11,596	4981	0.8366
	N86Y	2.165	3.377	0.4605

*P* value is significant when less than 0.05

*GM*: geometric mean, *IC*_*50*_: half maximal inhibitory concentration, *WA*: wild allele, *MA*: mutant allele

## Discussion

It is essential to have effective anti-malarial drugs to fight malaria. Artemisinin combination therapies were introduced as first-line therapy in this context. However, growing resistance to ACT has been observed in Southeast Asia: in Cambodia in 2006, Myanmar and Thailand in 2008, and Vietnam in 2009, and Laos in 2013 [15]. The rationale for monitoring resistance phenotypically by the in vitro method is that several anti-malarial drugs can be tested at the same time, and the evolution of the sensitivity or resistance of parasite populations to drugs either in use or no longer in use can be studied. The study of molecular markers of resistance informs the level of resistance of the *Plasmodium* population to drugs at the genetic level. This will result in better understanding of which drugs to monitor in vivo, which combinations to avoid, and those that can be used effectively for the management of malaria. In Senegal, pyrimethamine combined with sulfadoxine is used for intermittent preventive treatment for pregnant women. Further SP, plus amodiaquine is used for preventive seasonal treatment for children under 5 years old in areas with high transmission of malaria [16]. Chloroquine was eliminated in Senegal in 2003 following cases of resistance in vivo [17]. Piperaquine combined with dihydroartemisinin is used for the third-line treatment of non-complicated malaria. For molecular markers of resistance, the mutation on codons K76T of *pfcrt* gene and N86Y of *pfmdr1* has been demonstrated to be associated with resistance to chloroquine [18–22]. Resistance to amodiaquine is associated with the N86Y mutation and cases of cross-resistance between amodiaquine and chloroquine have been observed. For pyrimethamine, the mutation on codon S108N is strongly associated with resistance [23]. In vivo resistance of *P. falciparum* to chloroquine has been confirmed in Pikine, Moulomp (Casamance) and Fatick [24]. Indeed, the emergence of resistance to chloroquine in Senegal were reported in 1988 in Dakar with 5.7% therapeutic failure [25]. These cases then increased to 47.5% in 1990 and 25–30% in 1992 in Pikine [26], leading to the withdrawal of chloroquine for treatment of non-complicated malaria in Senegal in 2003. However, amodiaquine, which has some cross-resistance with chloroquine, is always used in combination for the treatment or prevention of malaria. In Dakar in 2010 a geometric mean of 41.63 nM for chloroquine and 19.4 nM for amodiaquine was found with another ex vivo technique [13]. At Pikine, an in vitro sensitivity study conducted in 2000 showed 31% of resistance to chloroquine with a geometric mean of 272 nM [27]. In 2001, a geometric mean of 135 nM was registered [28]. Prevalence of mutation of 51% on codon K76T of *pfcrt* and 11% on the N86Y of *pfmdr1* gene was recorded in 2014.

The prevalence of the 76T allele in isolates from Pikine was 72.4% when chloroquine was used (2000–2003), 47.16% during the period of the use of amodiaquine-SP for first-line treatment (2004–2005) and 59.46% with ACT used between 2006 and 2009. N86Y mutation had decreased between 2005 and 2009 and it was about 20% in 2009 [29]. A selection of N86 and K76 alleles were noted in Thiès, another region in Senegal, in 2013 [30]. The results of this study have shown that the mutation on codon N86Y was related to the decrease in sensitivity to chloroquine. For amodiaquine, the geometric mean of the isolates with the mutation N86Y was higher compared to isolates with wild-type allele, but the difference was not significant. For piperaquine, no relationship between genotype and IC_50_ was observed. An association was found between the presence of the 76T allele and the decrease in sensitivity to chloroquine (p = 0.0195) but not to amodiaquine (0.0539) and piperaquine (0.9370). A decrease in ex vivo sensitivity and an increase in the prevalence of the N86Y mutation relationship was not significantly found with amodiaquine (p = 0.5290) and the geometric mean for piperaquine was very low compared to that found in other countries [30–34]. These compounds are currently used in combination with dihydroartemisinin for piperaquine, SP and artesunate for amodiaquine. Good ex vivo sensitivity of isolates to dihydroartemisinin was found, implying continued effectiveness of one of the partner drugs of ACT used in the treatment of non-complicated malaria in Pikine.

Used since 2003 in Senegal, first as temporary replacement of chloroquine for the treatment of non-complicated malaria, SP is now used for preventive treatment of malaria. The results showed low ex vivo sensitivity of isolates to pyrimethamine. This was accompanied by a high prevalence of mutations in codons N51I, C59R and S108N of the *pfdhfr* gene. A strong presence of mutation on codon S108N (67 and 24%) and 51/59 (40 and 20%) were recorded, respectively, for Thiès in 2003 and Pikine in 2002. At Pikine, 65% (N51I), 61% (C59R) and 78% (S108N) of mutation was found [35]. These results suggest that resistance to pyrimethamine emerged before the introduction of the SP association. The double mutation 437/540 of the *pfdhps* gene has been demonstrated as being related to resistance to sulfadoxine [10]. Ex vivo sensitivity to sulfadoxine of isolates has not been studied. However, an absence of mutation on codon K540E and high prevalence of mutation at codon G437A/T has been recorded, which confirms the efficacy of sulfadoxine. On the other hand, studies revealed that triple mutation 108–59–51 is strongly associated with resistance to SP in African isolates [36] and that the presence of the double mutation 437/540 indicates a high risk of treatment failure in SP [9]. An increase of triple mutation and quadruple mutation in Pikine was noted [35], but the quintuple mutation was absent. However, mutation of 2.12% at codon 540 in *pfdhfr* was found in Dakar [36].

## Conclusion

Monitoring the sensitivity of *P. falciparum* populations to anti-malarial drugs is a necessity for effective malaria case management. An increase in the sensitivity of isolates to chloroquine Good efficacy of dihydroartemisinin amodiaquine and piperaquine and decrease in sensitivity to pyrimethamine were observed.

## Abbreviations

SP: sulfadoxine–pyrimethamine
ACT: artemisinin-based combination therapy
Pfdhps: *Plasmodium falciparum* dihydropteroate synthetase
Pfdhfr: *Plasmodium falciparum* dihydrofolate reductase
WHO: World Health Organization
ICEMR: International Centres of Excellence for Malaria Research
DMSO: dimethyl sulfoxide
RPMI medium: Roswell Park Memorial Institute medium
HRM: high resolution melting
DNA: deoxy ribo nucleic acid
PCR: polymerase chain reaction
Pfcrt: *Plasmodium falciparum* chloroquine resistance transporter
Pfmdr1: *Plasmodium falciparum* multidrug resistance protein 1
CQ: chloroquine
AMQ: amodiaquine
PQ: piperaquine
DHA: dihydroartemisinin
